# Prolonged intestinal transit and diarrhea in patients with an activating *GUCY2C* mutation

**DOI:** 10.1371/journal.pone.0185496

**Published:** 2017-09-28

**Authors:** Hilde L. von Volkmann, Ingeborg Brønstad, Odd Helge Gilja, Rune R. Tronstad, Dag Andre Sangnes, Ragnar Nortvedt, Trygve Hausken, Georg Dimcevski, Torunn Fiskerstrand, Kim Nylund

**Affiliations:** 1 National Centre for Ultrasound in Gastroenterology, Haukeland University Hospital, Bergen, Norway; 2 Department of Clinical Medicine, University of Bergen, Bergen, Norway; 3 Department of Pediatrics, Haukeland University Hospital, Bergen, Norway; 4 Department of Clinical Science, University of Bergen, Bergen, Norway; 5 Centres for Medical Genetics and Molecular Medicine, Haukeland University Hospital, Bergen, Norway; University of Nevada School of Medicine, UNITED STATES

## Abstract

**Introduction:**

Increased intestinal hydration by activation of the epithelial enzyme linked receptor guanylate cyclase C (GC-C) is a pharmacological principle for treating constipation. Activating mutations in the *GUCY2C* gene encoding GC-C cause Familial *GUCY2C* diarrhea syndrome (FGDS) which has been diagnosed with severe dysmotility.

**Aim:**

To investigate gut motility and hormones before and after a meal in FGDS patients and compare with healthy controls (HC).

**Subjects and methods:**

Bristol stool chart and stool frequency was assessed. Before and after a meal occlusive and non-occlusive contractions were obtained using ultrasound. A wireless motility capsule (WMC) recorded gut transit time, pH, contractions and pressure. Plasma levels of selected gut hormones were measured at different time points.

**Results:**

The FGDS patients had 4 (range 1–10) loose stools/day and prolonged total gut transit time compared to HC, 55.5 h vs 28.5 h, respectively,with significantly increased colon transit time. In FGDS patients, pH in duodenum, small bowel and colon was increased and the number of contractions and the intraluminal pressure were significantly decreased, measured by WMC. Ultrasound showed in small bowel increased number of non-occlusive contractions in the FGDS patients. Serotonin (5-HT) plasma levels in the HC peaked 30 min after the meal, while the FGDS patients had no response.

**Conclusion:**

Despite having diarrhea, the FGDS patients have prolonged transit time through the gut compared to HC, particularly in colon. The reduced number of intestinal contractions and lack of 5-HT release after a meal in FGDS patients surprisingly resemble colonic motility disturbances seen in patients with constipation.

## Introduction

The guanylate cyclase C receptor (GC-C) in humans is expressed on the epithelial cells from duodenum to rectum [1], and the receptor is activated by the endogen ligands guanylin (GN) and uroguanylin (UGN), and the heat stable ST toxin from *E*.*Coli* as well as the drug linaclotide used in the therapy of irritable bowel syndrome with constipation (IBS-C) [2,3]. The GC-C pathway is important for controlling the systemic fluid and electrolyte homeostasis [1]. Activation of GC-C leads to increased secretion of chloride, sodium and water to the intestinal lumen [4]. Apart from intestinal hydration, GC-C signalling influences the intestinal barrier function and epithelial differentiation, proliferation and tumorigenesis. A role in regulation of satiety and lipolysis has also been suggested [5–8].

In 2012 Fiskerstrand *et al* described an activating mutation of *GUCY2C* encoding for GC-C leading to the Familial *GUCY2C* diarrhea syndrome (FGDS) in 32 members of a Norwegian family [2]. The clinical characteristics of this rare, autosomal dominant inherited disease are diarrhea, intestinal inflammation (25% have Crohn’s disease (CD)) and symptoms resembling IBS with diarrhea (IBS-D), but without severe abdominal pain. Thirty percent of the family members had surgery due to small bowel obstruction, which in some was due to volvulus and pseudo-obstruction [2]. Chronic activated GC-C signalling in FGDS patients leads to elevated intracellular cyclic guanosine monophosphate (cGMP) leading to phosphorylation of the cystic fibrosis transmembrane regulator (CFTR) resulting in increased secretion of Cl^-^, bicarbonate and fluid into the intestinal lumen. GC-C activation also leads to an inhibition of the Na^+^/H+-exchanger followed by reduced absorption of Na^+^[9].This leads to increased efflux of fluid into the intestinal lumen explaining the diarrhea seen in these patients. In a recent study, we used ultrasound to examine the fluid distribution and motility, and found that the FGDS patients had severe dysmotility in the intestine, particularly in the small bowel [10]. However, very little is known about how a chronic activated GC-C signalling is affecting the human intestine, and in this study we aimed to elucidate this.

Wireless motility capsule (WMC, SmartPill Corporation, Given Imaging, Yokneam, Israel) and ultrasound are two different methods to study the intestinal motility. The WMC can be used to measure total gut transit time, regional gut transit time through the stomach, small bowel and colon, as well as temperature, pH, contractions per min and intraluminal pressure through the whole gut. WMC recordings are comparable with established motility testing modalities and is mainly used to diagnose gastric emptying, particularly in patients with diabetic gastroparesis [11]. Furthermore, it is also suggested in the diagnosis of constipation and various motility disorders of the gut [11,12]. Ultrasound is a non-invasive tool for bowel examination, gives us the possibility to examine the intestinal contractility pattern in real time, and can be used to observe aspects of contractility which are not detected by methods relying on pressure differences only [13–17].

Dysmotility in the intestine is defined as an inadequate (incoordinated or excessive response) in the intestinal muscular activity [18], of exogenous or endogen origin, *e*.*g*. myopathic or neuropathics disorders [19]. Motility disorders may manifest differently, and both IBS-C and IBS-D have been proposed to involve motility disturbances.

Several gastrointestinal (GI)- hormones have been shown to influence hydration and GI motility such as GN, UGN and serotonin (5-HT), and the ileal brake regulating hormones glucagon-like peptide-1 (GLP-1) and the pancreatic peptide YY (PYY). The “ileal brake” is mediated neuronally by sympatho-adrenergic and vagal pathways and hormonally by gut peptides (GLP-1, PYY and neurotensin) exerting an inhibitory reflex affecting the transit time of luminal content through the gut [20]. Gut hormones are mainly stored in the enteroendocrine cells (EEC) which are found scattered throughout the intestine [21]. EEC cells act as sensors for intestinal luminal content (*e*.*g*.nutrients and bacterial products) enabling a cross-talk between the endocrine cells, immune cells, neurological and muscular system. Therefore, they are considered important for the regulation of intestinal motility [21].

In this study, we combined WMC and ultrasound with blood samples of motility regulating hormones, and hypothesized that FGDS patients have changed transit time, intraluminal pressure and pH in the gut, as well as changes in circulating levels of gut hormones.

## Subjects and methods

### Study participants

This is an explorative case control study. The controls were matched for age and gender. The majority of the FGDS patients live in western part of Norway, and 24 adults (> 18 years) were invited to participate in this study.

The HCs were recruited using bulletin boards at Haukeland University Hospital and via a website for subject recruitment.

The participants of the HC group were interviewed before inclusion and had no previous history of gastrointestinal disease. Body mass index (BMI) and blood samples (haemoglobin (Hb), c-reactive protein (CRP), creatinine, electrolytes, aspartate and alanine aminotransferases (AST and ALT) and glutamyltranspeptidase (GGT) were obtained.None of the HC fulfilled the Rome III criteria for IBS. Exclusion criteria were history of bowel surgery (except appendectomy) or gastrointestinal complaints or symptoms resembling IBS. None of the participants were allowed to use antidepressants or medications which could affect motility.

### Preparation prior to the examination

Three days prior to the examination all participants had to avoid food rich on tryptophan such as nuts, chocolate, ice, large meals with beef or seafood. Alcohol or caffeine-containing products were prohibited 24 hours before the study and they had to refrain from alcohol as long as the capsule was in the gut. Samples from all participants were obtained after an overnight fast of >8 hours. All participants met in a fasting state on the examination day.

Bristol stool chart (BSC) was assessed in all participants, and shows seven categories of stool type where 1–2 indicate constipation, 3–4 normal and 5–7 indicate diarrhea[22] Only in the FGDS group additional information about stool frequency for the last 3 months and 24 hours was obtained. As the patients have symptoms resembling IBS-D, but without severe pain, we used the Irritable Bowel Symptom Score (IBS-SSS) in order to obtain the severity of their complaints [23].

Informed consent was obtained from all participants and the study was approved by the Regional Committee for Medical Research Ethics, Haukeland University Hospital (Registration number 2014/2222) and performed in accordance with the Declaration of Helsinki.

### Meal

A standardized meal called SmartBar (SmartPill Corporation, Given Imaging, Yokneam, Israel) was used in the study. It contains 260 kCal (fat 2 g, sodium 300 mg, carbohydrate 50 g, fiber 2 g, protein13 g), and was given 10 minutes prior to ingestion of the WMC.

### Wireless motility capsule (WMC)

The WMC system (SmartPill Corporation, Given Imaging, Yokneam, Israel) consists of an ingestible single-use capsule, a receiver and display software. The capsule measures 26 mm × 13 mm and has sensors that measure pH, temperature and pressure. The pH measurement is accurate to ± 0.5 pH units and pressure measurement is accurate to ± 5 mmHg below 100 mmHg. The gastric emptying time (GET) is defined as time between capsule ingestion and entry to pyloric passage (abrupt and sustained rise in pH (> 2 units)). Small Bowel transit time (SBTT) is defined as the time between passage into the small bowel and to a drop in pH of approximately 1 unit indicating the arrival of the capsule in the cecum [24]. Colon transit time (CTT) is the time from cecum until body exit (BE) when s a major drop in temperature is seen.Small bowel and colon transit time (SBCTT) and the whole gut transit time (WGTT) are recorded. According to the software specification,pressure and pH in duodenum are defined as the first 30 min after gastric emptying.

The test started with the ingestion of a SmartBar after an overnight fast. After 10 min the patients swallowed the WMC with 120 ml of water. After intake of the capsule the participants were not allowed to eat the following 6 hours, but could drink up to 100 ml water.

### Ultrasound

The ultrasound examination was conducted with a Logic E9 ultrasound scanner (GE Healthcare, Milwaukee, USA), using a convex probe C1-5 (2.8–5.0 MHz) for abdominal overview and a linear probe 9L (8.4–9.0 MHz) for detailed bowel examination at baseline (before intake of the meal). All ultrasound measurements were performed with patients lying in a prone position. The following procedures were repeated 30, 60, 120 and 240 min after intake of the meal.

Occlusive and non-occlusive contractions were counted and defined as in our previous study [10] where an occlusive contraction was defined as an active indentation of the bowel wall occluding the lumen, and a non-occlusive contraction as an active indentation in which luminal content can be observed between the anterior and dorsal wall during a contraction. We obtained one minute video recordings from the jejunum in the upper left quadrant and of the ileum in the lower right quadrant. If the lumen was not fluid-filled, we were not able to differentiate between occlusive and non-occlusive contractions, and the measurements were excluded.

The number of fluid-filled small bowel loops was counted using a magnetic navigation system as shown in our previous study [10] where we used a commercially available magnetometer-based position and orientation measurement (POM) device on the ultrasound scanner which enabled tracking of the scan head in the magnetic field.

Presence of small bowel segments with turbulent intraluminal and back and forth flow of content and non-occlusive contractions with no propulsive movements were defined as “snowglobe” phenomenon [10], and noted as present or not in left upper quadrant and lower right quadrant, respectively.

The antrum was localized on a sagittal section in front of the superior mesenteric vein and the abdominal aorta. The image was frozen, and the area measured by tracing the circumference following the outer edge of the hypoechoic proper muscle layer using the built-in caliper of the scanner. In order to study if there were differences in the mesenteric flow in fasting subjects and in response to the meal, we examined the resistive index (RI) of the superior mesenteric artery (SMA). This reflects the circulatory resistance in the small bowel and proximal large bowel[25] It was sampled with the SMA oriented in long axis in the sagittal plane 2 cm distal to its origin to avoid turbulence. The angle between the ultrasound beam and the SMA was kept smaller than 60°. The RI was calculated according to the formula: peak-systolic velocity minus end-diastolic velocity divided by the peak-systolic velocity.

### Blood samples

Gastrointestinal hormones (ProUGN, ProGN, 5-HT, PYY and GLP-1) were measured before (baseline) and after the meal (30, 60, 120 and 240 min). Electrolytes, CRP, creatinine, and electrolytes were measured at baseline. The blood was taken from a peripheral vein using 18G cannula, extension line and a three ways stop cock. The system was flushed with 2 ml sterile saline after extraction.

Plasma for ProGN, ProUGN and PYY determination was obtained from EDTA blood by centrifugation at 1800 x g for 10 min at 4°C. Trasylol^™^ (Bayer Pharmaceuticals) was added to the blood samples for PYY analyses immediately after blood collection to give a final concentration of 500 KIU/mL Trasylol^™^. Plasma for GLP-1 determination was obtained by adding 10 μL DPP-IV inhibitor (Dipeptidyl peptidase IV, DPP4-010, DRG Diagnostics, Germany) per mL EDTA blood prior to sampling, and centrifuged at 1000 x g for 10 min at 4°C. The blood samples were put on ice immediately after sampling and centrifuged within 20 min. The plasma was then aliquoted and stored at -80°C until analysis.

Concentration of 5-HT was measured in platelet poor plasma (PPP). To prepare PPP, 3 mL mL EDTA blood was centrifuged at 200 x g for 10 min at room temperature within 5 min from sampling to give platelet rich plasma (PRP). Furthermore, 400 μL of the upper two-thirds of the PRP was immediately transferred to another tube and centrifuged at 4500 x g for 10 min at 4°C to give PPP. The PPP was aliquoted and stored at -80°C until analyzed for 5-HT.

### Hormone analyzes

The plasma concentrations of the gastrointestinal hormones were determined by commercial enzyme linked immunisorbent assays (ELISA) kits. ProGN and ProUGN were analyzed with kits from BioVendor, Karasek, Czech Republic (catalog number RD191069200R and RD191046100R, respectively). GLP-1 and 5-HT were determined with kits from IBL International, Hamburg, Germany (catalog number RE53121 and RE59121, respectively) and total PYY was determined using a kit from EMD Millipore Corporation, Billerica, MA, USA (catalog number EZHPYYT66K). The ELISAs were performed according to the manufacturer’s instructions. The optical density from each ELISA was measured by SPECTRAmax microplate reader (Molecular Devices, Sunnyvale, CA, US) and concentrations of each specimen were calculated by the SoftMaxPro Software version 5.4.5.000 (Molecular Devices, Sunnyvale, CA, US) using an algorithm for the best standard curve fitting.

### Statistics

Data are shown as median and range. Differences between groups were measured using the unpaired t-test or Mann-Whitney U-test. Pearson or Spearman was used for analyses of correlation. The non-parametric methods were used when variables were not considered to have a normal distribution. P-value < 0.05 was considered significant. Interobserver agreement was assessed for all 38 WMC examinations and was analyzed by two different observers (HLvV and DAS). Limits of agreement using the difference between observations versus mean values of the two observations were calculated according to Bland and Altman [26]. Data were analysed with SPSS 22.0 Software (Chicago, Illinois, USA), figures were made with both SPSS and Graph Pad Prism version 5.02 for Windows (Graph Pad Software Inc., La Jolla, CA, USA).

## Results

Twenty-one FGDS patients between 21 and 77 years, (median 49 years), 10 females and 11 males, and 24 healthy controls (HC) between 21 and 76 years,(40 years), 14 females and 10 males, participated in this study.

### Clinical characteristics of gastrointestinal functions

The majority of the participating FGDS patients had loose stools on daily basis(n = 20) and prior to the FGDS diagnosis they were suspected of having IBS-D. Seventeen patients reported having loose stools from childhood. Six patients had undergone abdominal surgery, and 5 patients had been diagnosed with CD. Only one FGDS patient reported no abdominal discomfort.

The median number /24 hours of stools for FGDS patients were 4 (range 1–10) and 4 (1–6) during the last 3 months. 80% of the FGDS patients had watery or liquid stool with BSC 7 (4–7), wheras all the healthy controls had normal stool concistency 4 (3–4). Therefore, the number of stools per day were not recorded in the HC The median IBS-SSSwas only obtained in the FGDS patients, and was 132 (30–335) which corresponds to scores observed in patients with mild IBS [27]. All the HCs had normal clinical examination and blood samples. BMI in FGDS was 24 (19–29) and in HC 24 (19–30) respectively.

### Gut transittime and pH

Two FGDS patients refused the WMC examination, and in one patient the recordings were incomplete due to failure of the device. Four of the FGDS patients had contraindications for the examination due to previous multiple bowel resections and symptoms of intermittens partial/pending bowel obstruction. One HC had incomplete signals from the colon due to device failure, and the pH, pressure and transit time in colon were excluded from the analysis. Finally, 24 HC and 14 FGDS patients were examined with WMC.

**Fig 1:**
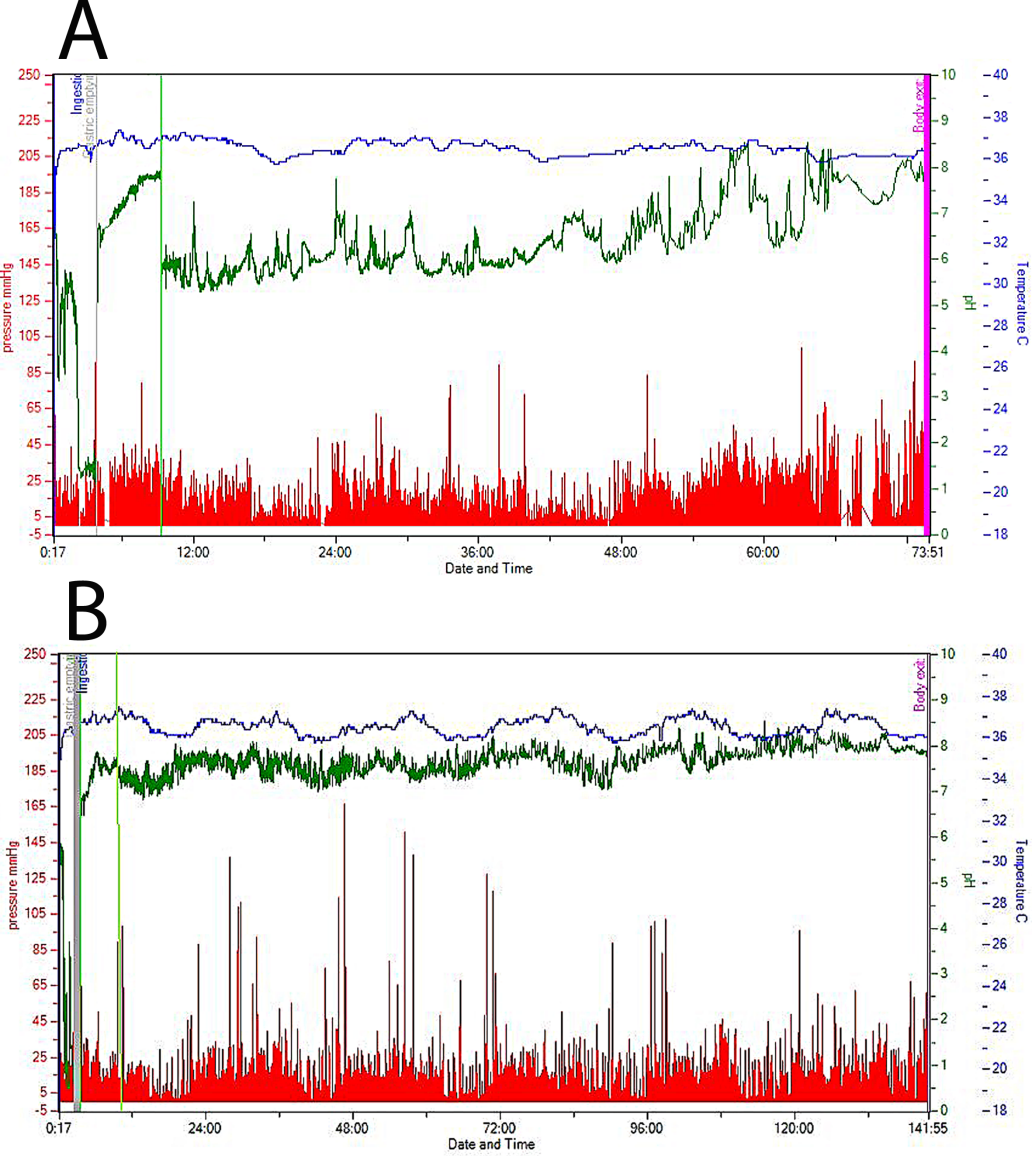
A summary of WMC (wireless motility capsule) data. The blue line represents the temperature, the green the pH and the red peaks the pressure along the entire gut. The green vertical lines display the ileocoecal transition zone. In 1A (Healthy Control) the pH drop is more than 1 unit, in 1B (FGDS) the characteristic pH drop in the ileocoecal junction is not present. This was found in 6 FGDS patients.

The transit time for the whole gut and colon was significantly longer in the FGDS patients than in the HC (p< 0.01)(Table 1). In six of the FGDS patients, the expected pH drop when the capsule reached the cecum was not observed, and the transition was determined by observing changes in the variability of the pressure waves and pH (Fig 1). There was also a trend towards longer transit time through the small bowel in the FGDS patients 6 h (2.3–47.4) compared to the healthy group,4.2 h (2.6–6.6.), (p = 0.07).

**Table 1:** Transit time. Transit parameters in hours, (median and range) for Gastric Emptying (GET), Small Bowel (SBTT), Colon (CTT), Small Bowel and Colon (SBCTT) and Whole Gut Transit Time (WGTT) for Healthy Controls and FGDS patients.

	Healthy controls		FGDS patients		
Segments	Median (h)	Range	Median (h)	Range	p-value
GET	3.0	0.6–5.2	3.1	2.0–5.7	0.4
SBTT	4.2	2.6–6.6	6.0	2.3–47.4	0.07
CTT	21.5	3.1–64.6	39.5	20.9–137.4	<0.01
SBCTT	25.6	6.6–70.1	50.8	26.1–141.3	<0.01
WGTT	28.5	9.3–73.6	55.4	27.6–143.4	<0.01

**Fig 2:**
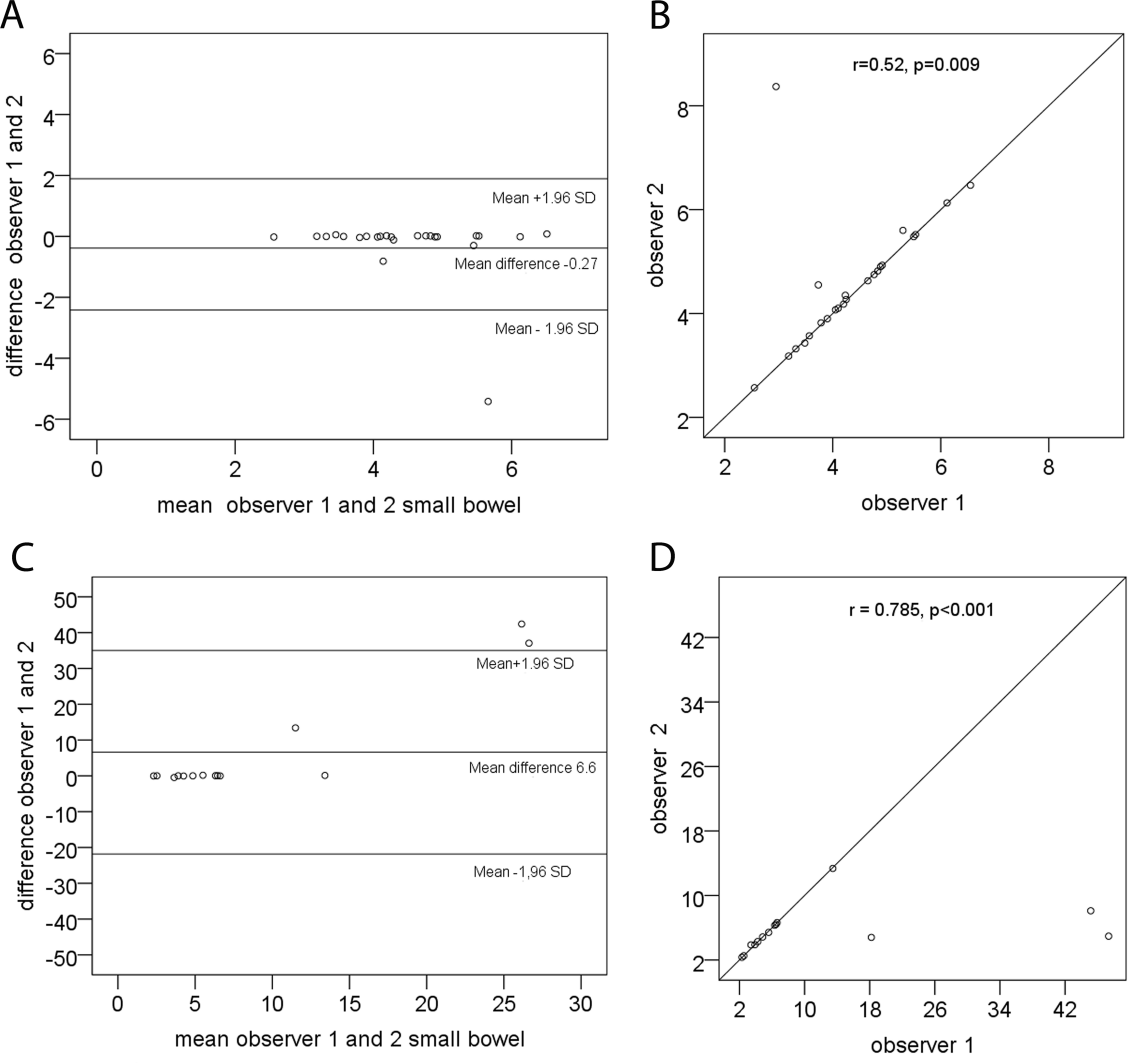
Interobserver analyses from small bowel. Scatter plots and Bland-Altman plots with limits of agreement depicting bias and error between observer 1 and observer 2 for detection of small bowel transit time recorded with wireless motility capsule in healthy controls (2A,B) and in patients with Familial *GUCY2C* Diarrhea Syndrome (2C,D).

The results of the interobserver analyses from all gut segments in FGDS and HC are displayed in S1 Table. The small bowel comparison is displayed in Fig 2 as correlation plots and Bland & Altman plots.

**Fig 3:**
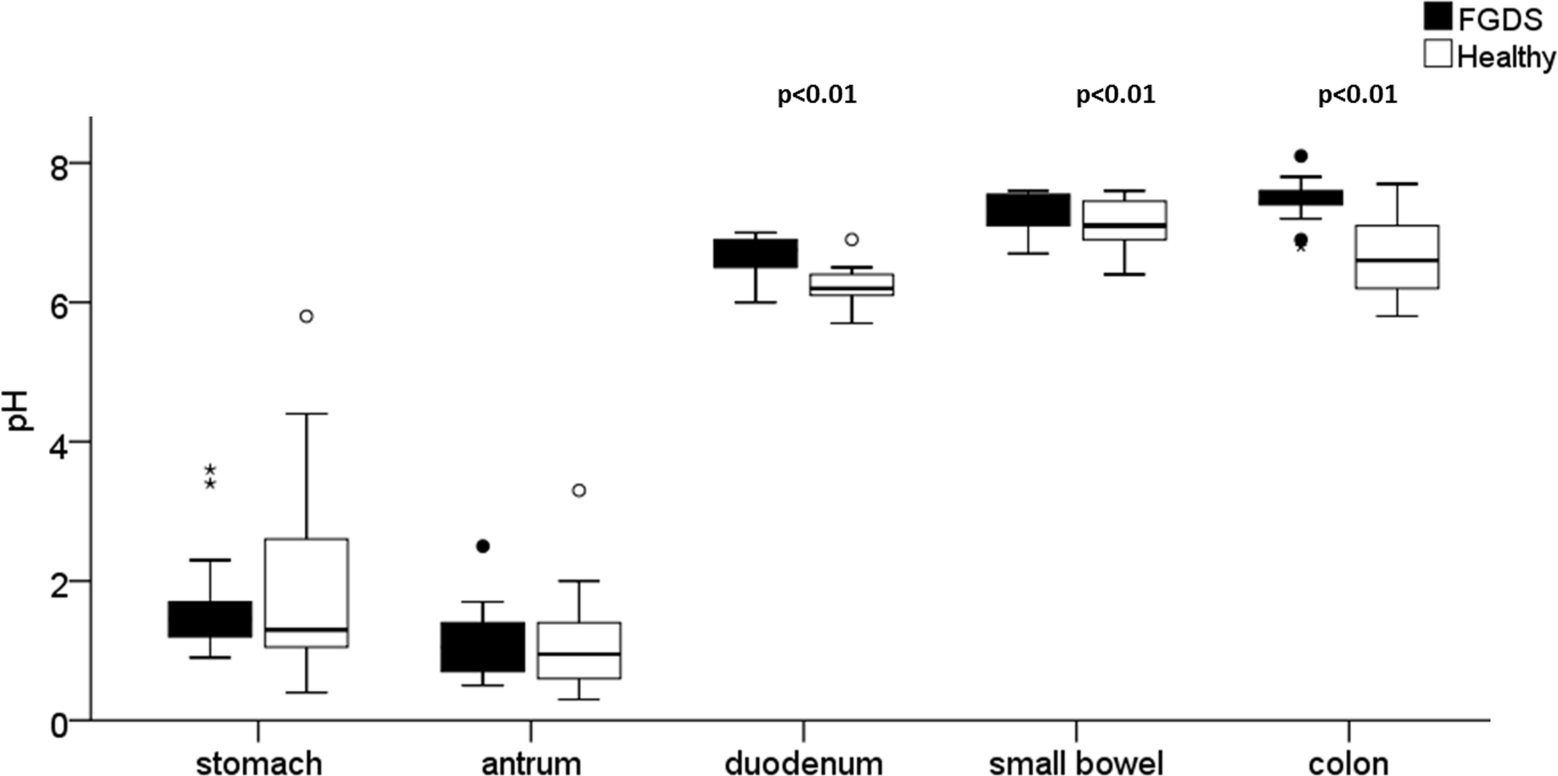
Gastrointestinal pH measurements. Box plots showing the elevated pH values in the duodenum, small bowel and colon found in patients with Familial *GUCY2C* diarrhea syndrome (FGDS) compared to healthy controls (HC). There was no difference in the stomach and antrum. The median is shown within the box; the box represents the 25 and 75 percentiles and the whiskers 10 and 90 percentiles.

There were no significant differences in the pH between FGDS and HC in the stomach and antrum (p = 0.85, p = 0.78), but the pH was significantly elevated in duodenum (p = 0.009), in small bowel (p = 0.007) and colon (p = 0.001) in FGDS patients compared to HC (Fig 3).

### Intraluminal pressure and intestinal contractions

Intraluminal pressure measured by WMC was significantly lower in FGDS patients compared to HC in duodenum (p = 0.01), and in small bowel (p = 0.02) and in colon (p = 0.002), whereas in antrum and stomach there was no significant differences (p = 0.41, p = 0.33) (Table 2).

**Table 2:** Pressures obtained by Wireless Motility Capsule (WMC). Intraluminal pressure parameters in mmHg, median and range obtained with WMC. Colon data from 23 healthy controls and 14 patients are displayed.

	Intraluminal Pressure in mmHg				
	Healthy controls		FGDS patients		
Segments	Median	Range	Median	Range	p value
Stomach	2.3	1.4–3.4	2.0	1.4–3.4	0.41
Antrum	2.6	1.4–6.8	2.6	1.1–4.6	0.33
Duodenum	3.1	2.0–5.6	2.4	1.6–3.6	0.01
Small Bowel	3.6	3.0–7.2	2.8	1.8–5.8	0.02
Colon	3.9	2.2–6.1	2.7	2.1–4.3	0.002

**Fig 4:**
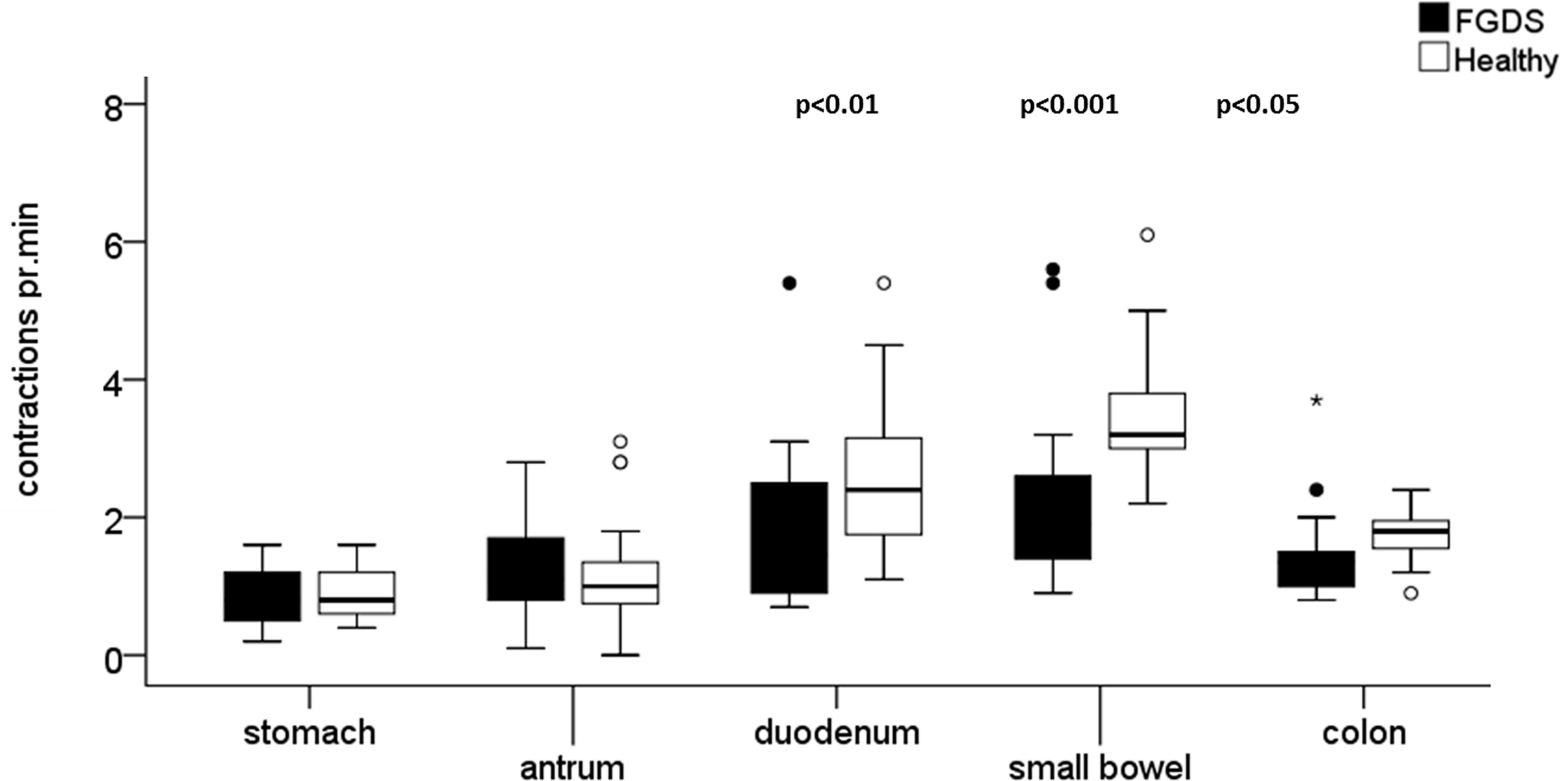
Number of contractions obtained by Wireless Motility capsule (WMC). Box plots showing number of contractions per min in stomach, antrum, duodenum, small bowel and colon recorded by WMC. There was no significant difference between the two groups in stomach or antrum. In the duodenum, small bowel and in colon there was significant differences between FGDS and HC. The median is shown within the box; the box represents the 25 and 75 percentiles and the whiskers 10 and 90 percentiles.

Correspondingly, there were significantly less contractions per min registered by WMC in duodenum (p = 0.03), small bowel (p = 0.001) and colon (p = 0.02) respectively in the FGDS patients compared with the HC, whereas in antrum and stomach there were no differences (p = 0.45, 0.46), Fig 4.

**Fig 5:**
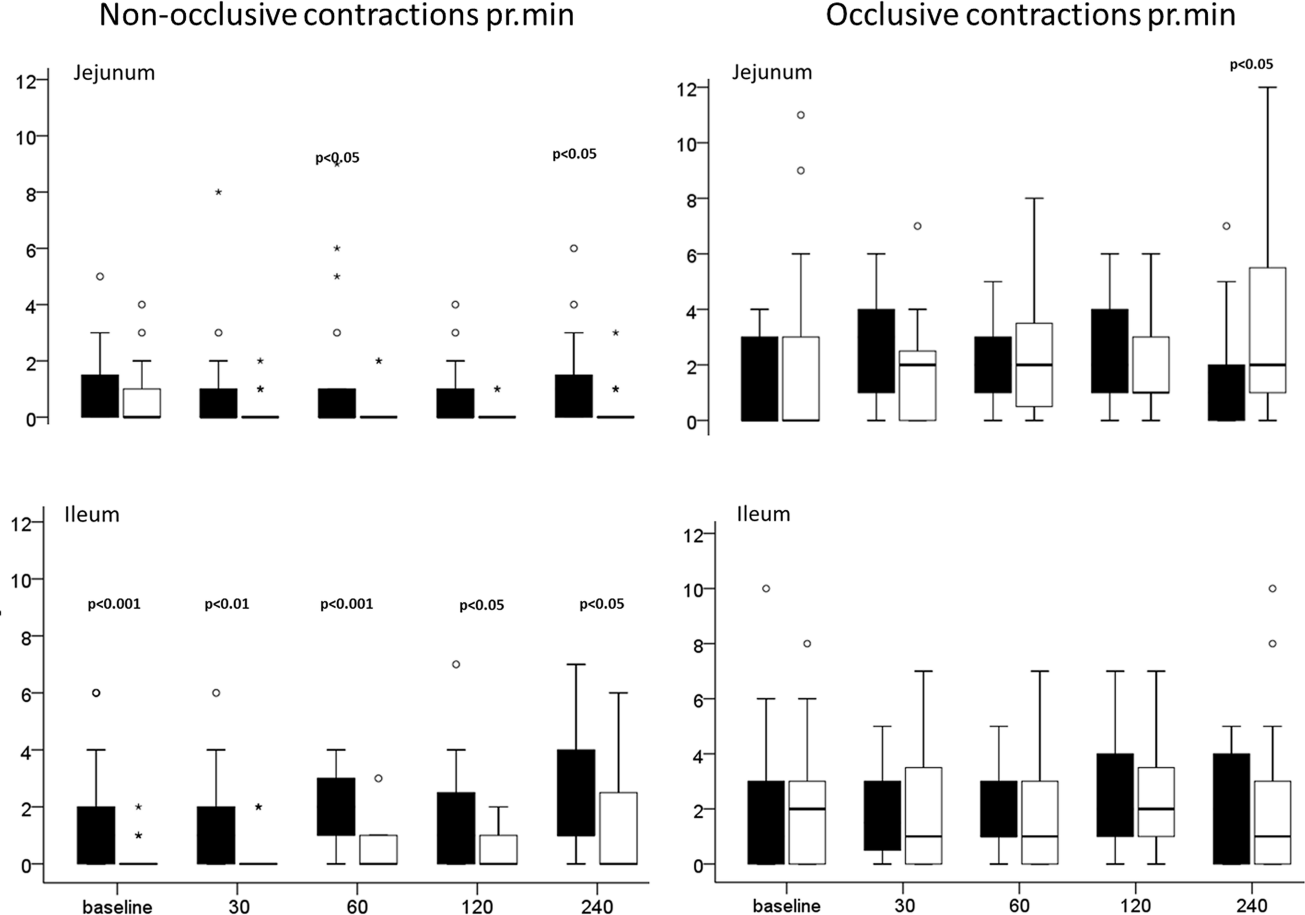
Contractions in small bowel obtained by Ultrasound. Box plots showing the number of non-occlusive ileal and jejunal contractions and the number of occlusive ileal and jejunal contractions pr min obtained by ultrasound for each of the study time points. The median is shown within the box; the box represents the 25 and 75 percentiles and the whiskers 10 and 90 percentiles. The black boxes display FGDS patients, the white boxes the Healthy controls.

Using ultrasound, significantly more non-occlusive contractions in the ileum were observed in the FGDS patients compared to HC at all measured points in time (Fig 5), but no differences in the number of occlusive contractions between the two groups at any time point. The jejunal non-occlusive contractions were significantly more in the FGDS patients at time points 60 and 240 min after the meal, Fig 5.

### Intestinal fluid content

**Fig 6:**
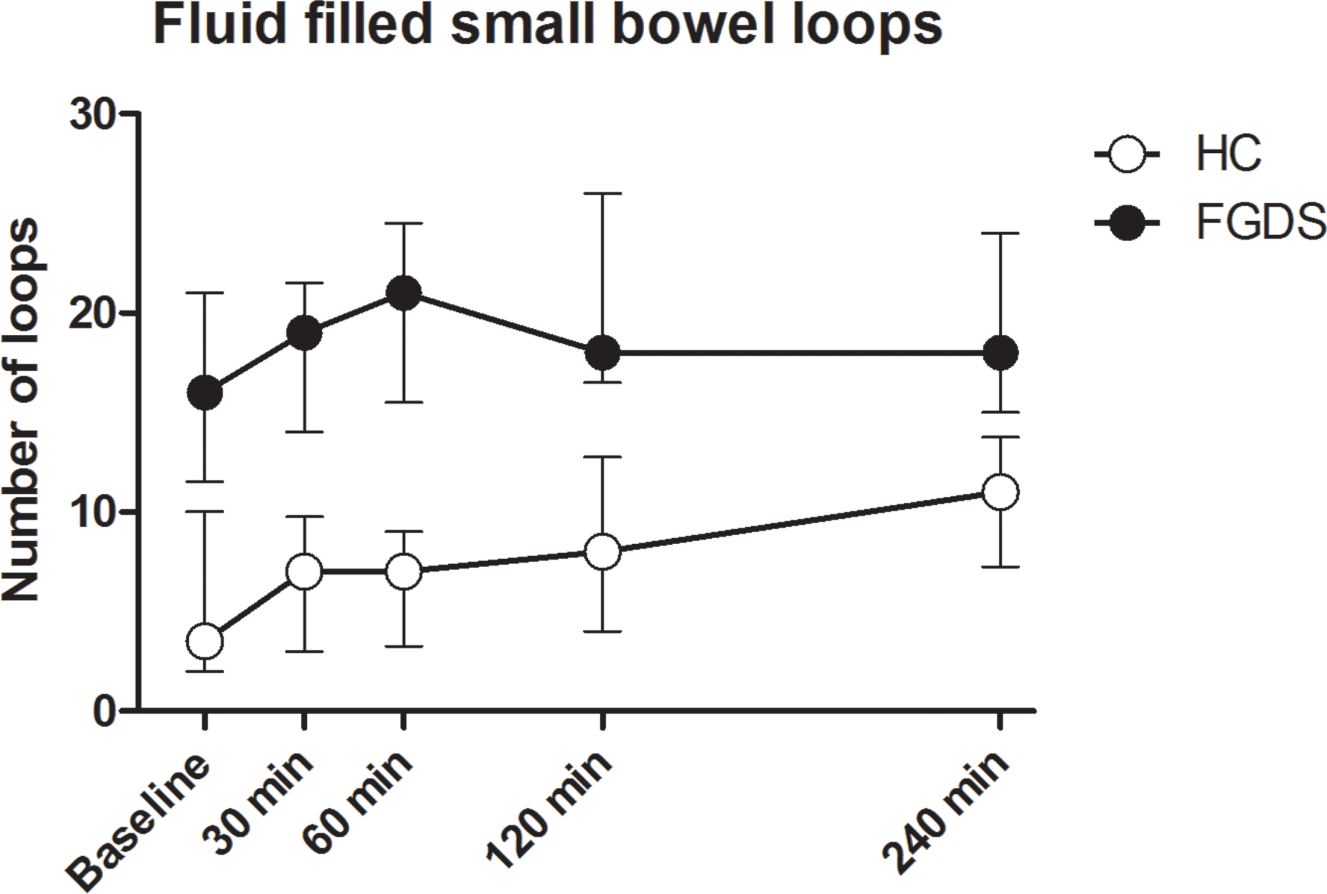
Fluid- filled small bowel loops. The number of fluid-filled small bowel loops obtained by ultrasound.There were significant differences between FGDS and HC for all measured points (p<0.001). Error bars are shown as median with IQR (25 and 75 percentiles).

The total number of fluid-filled small bowel loops was significantly increased at all measured points in time (p < 0.001) in FGDS patients compared to the HC (Fig 6).

“Snowglobes” were present in the jejunum in 18 of 21 FGDS patients and in 1 of 24 HC, and in ileum in 20 of 21 FGDS patients and in 1 of 24 HC.

There were no significant differences between the antral areal in FGDS and HC before the meal (p = 0.93) or in any of the postprandial (30, 60, 120 or 240 min) ultrasound measurements (p = 0.65, p = 0.33, p = 0.80, p = 0.64 respectively). The RI index in SMA showed no differences between FGDS or HC group before (p = 0.7) or after a meal (p = 0.72, p = 0.74, p = 0.64, p = 0. 22, respectively).

### Hormones

Blood samples from one of the HC was taken by a 20G cannula and the 5-HT measurement from this subject was excluded because smaller needle size probably will affect the result by giving elevated 5-HT levels. In addition, hormone analyzes of two FGDS patients were missing due to technical problems with blood sampling.

**Fig 7:**
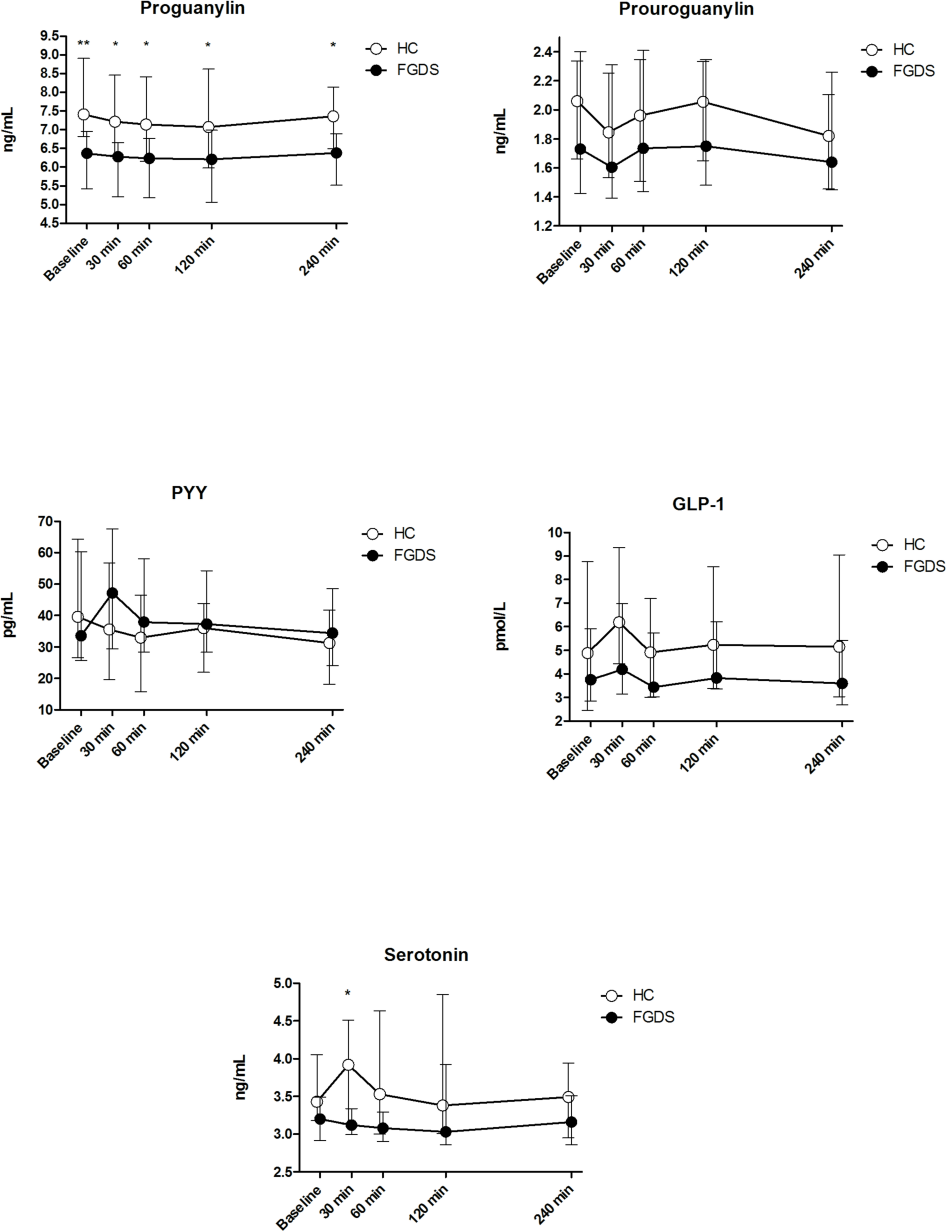
Gastrointestinal hormones. Median plots with IQR (25 and 75 percentiles) for the time course secretion of the different hormones before (baseline) and after a meal. Significant differences were found between FGDS and HC regarding plasma Proguanylin at all time points (baseline, 30 min, 60 min, 120 min and 240 min) and for plasma Serotonin 30 min postprandial (* p< 0.05,**p < 0.01). There were no significant differences in plasma measurements of GLP-1, PYY and Proguanylin between FGDS and HC.

Fig 7 illustrates the fasting and postprandial plasma hormone levels for all analyzed hormones in FGDS patients and HC in a time course. ProGN levels were significantly lower in FGDS patients compared with HC at baseline (p = 0.002) and all at postprandial timepoints (p<0.05), whereas 5-HT levels were significantly higher in HC 30 minutes after the meal compared to FGDS patients (p = 0.01).

There were no significant difference between FGDS patients and HC in plasma levels of ProUGN, GLP-1 and PYY.

### Correlations

#### Stool concistency (for both groups)

There was a weak correlation between stool consistency measured by BSC and CTT (r = 0.39, p = 0.02), and SBCTT (r = 0.5, p = 0.001), and WGTT (r = 0.47, p = 0.01), but no correlation between BSC and SBTT (r = 0,28, p = 0.2). A weak negative correlation between BSC and ProGN was seen (baseline; r = - 0.53, p< 0.001). None of the other hormones tested, 5-HT, ProUGN, GLP-1 or PYY showed correlation to stool consistency.

#### Stool frequency (only obtained in FGDS patients)

There were no correlations between the number of loose stools with any transit time measurements in the FGDS patients. Neither did the number of stools correlate with the level of any gastrointestinal hormones.

#### Contractions, pressure and transit time (for both groups) obtained by WMC

Correlation between colonic pressure and contractions (r = 0.667, p< 0.001), and between small bowel pressure and contractions was found (r = 0.919, p>0.001).

A negative correlation, was found between CTT and colon contractions pr min (r = -0.509, p = 0.001) and between CTT and colonic pressure (r = -0.37, p = 0.031). No correlation between SBTT and contractions / pressure was found.

There were no correlations between number of loose stools/BSC and pressure /contractions pr min in small bowel or colon.

## Discussion

The main findings in this study are prolonged transit time through the gut, colon in particular, and reduced luminal pressure in FGDS patients compared to a healthy control group matched for age and gender. The number of contractions in duodenum, small bowel and colon recorded with the WMC wasalso reduced in the FGDS patients. Interestingly,the FGDS patients have increased number of non-occlusive contractions in small-bowel measured with ultrasound, compared to HC. FGDS patients also have elevated pH in duodenum, small bowel and colon. These findings support our previous results suggesting intestinal dysmotility in patients with increased GC-C signaling [10]. There were few significant differences in gastrointestinal hormones between FGDS patients and HC, but plasma levels of 5-HT in the FGDS patients did not peak after the meal as observed in the HC.

CTT was almost twice as long in the FGDS patients compared to the HC(39.5 h in FGDS patients versus 21.5 h in HC). Both were within normal range, but the difference between the groups was significant. There are conflicting results regarding intestinal transit time both for IBS patients with constipation and for those with diarrhea probably reflecting the many different important regulatory factors affecting transit time and motility in the gut [11,28–30]. Prolonged CTT may be found in patients with slow transit constipation [31], but only a small range of patients with IBS-C has slow transit time [32–34].

In accordance with other studies, we found that stool frequency correlated poorly with the CTT [35,36]. Interestingly, we found a positive correlation between SBCTT, CTT, WGTT, respectively and stool consistency according to the BSC. Although the correlation was weak, these findings challenge the widely held notion that loose stools correlate with fast intestinal transit [37–39]. In addition, there was a weak negative correlation between CTT, colon contractions and colon pressure obtained by WMC. We also found a negative correlation between BSC and plasma proGN which has not been described before. ProGN is secreted from the epithelial cells both into the blood and intestinal lumen, and in conditions with loose stools it is possible that secretion of proGN is reduced in order to maintain the fluid homeostasis.

The findings with regards to contractions measured by WMC and ultrasound may seem contradictory. However, since the WMC only captures pressure inducing contractions it may not register the non-occlusive contractions recorded with ultrasound. The pathogenic mechanisms for the reduced and inefficient contractions in the FGDS patients are not clear, but it is unlikely to be related to grossly disturbed levels of gastrointestinal hormones, as only plasma ProGN was significantly lower in the FGDS patients compared to the HC at baseline and postprandial. Notably, the FGDS patients had no peak in plasma 5-HT secretion after the meal, as we could observe in the HC. This suggests similarities to IBS-C patients where studies show no peaks in plasma 5-HT after a meal [40]. 5-HT is an important neutrotransmitter in the gut, but the excact mechanism is debatable, as some suggest that 5-HT secretion in the gut is a result of peristalsis and not the cause of it [41,42].

The increased formation of cellular cGMP caused by activation of GC-C [2,9] could possibly influence smooth muscle contractions. cGMP is also transported out of the intestinal epithelial cell where it has been shown to affect sensory afferent nerves [43–46]. This may explain the lack of pain on daily basis observed in FGDS patients [2]. However the extracellular cGMP may have other effects as well. In the intestine the intracellular nitric oxygene/soluble Guanylate Cyclase pathway (NO/sGC) is important for the smooth muscle relaxation [47]. It has been shown in mice that extracellular cGMP can affect this pathway in cerebellar tissue [48]. If a similar effect occurs in the smooth muscle cells in the small bowel, this may cause a relaxing effect in intestinal smooth muscles which in turn could lead to dysmotility and ineffective contractions. This hypothesis needs to be addressed in future more mechanistic studies.

While the intestinal luminal pH is highly influenced by small chain fatty acids and other metabolites produced by the intestinal microbiota which in turn are dependent on the nutrition [49], our study demonstrates the influence on human intestinal pH by GC-C activation. The luminal content is more alkaline in FGDS patients compared to HC from duodenum and throughout the intestine. This is in accordance with the known effects of GC-C activation, facilitating secretion of bicarbonate and impeding sodium and proton exchange across the intestinal epithelium. Thus, increased intestinal pH is likely a primary effect of GC-C activation particularly in the smaller intestine which contains few bacteria, and is likely the reason why a drop in pH between the small intestine and the cecum was absent in 6 of the FGDS patients. However, FGDS patients have been shown to harbour a particular intestinal microbiota profile (*Tronstad et al*, in press) and this may additionally influence the pH in the colon. Wang *et al* (2015) found by comparing a Swedish and American population, that the Swedes exhibited lower pH in the gut generally and a longer SBTT and shorter CTT [50]. These findings may reflect differences in nutrition composition which may lead to differences in the microbiome, thus affecting the metabolism, fermentation and finally gut motility and transit time. An increase in the luminal pH of the intestine caused by GC-C activation might override the effect of bacterial metabolites on luminal pH and may play a role of the increased transit time observed in FGDS patients.

A limitation of this study is that we did not observe the typical pH drop from small bowel to colon in 6 of the FGDS patients and the transition was determined by the pressure waves and pH pattern (Fig 1). However, the interobserver analyses showed high limits of agreement, indicating that the recordings throughout the intestinal transit gave reliable results. Another limitation of this study is the length of the videos acquired during ultrasound examinations (1 min videos) which may not fully represent the spectrum of all small bowel movements. However, the patients were all examined for altogether 4 hrs. Also, all subjects had an overnight fast and received the same meal suggesting that they all were in similar motility states at least for the time points registered after the meal.

## Conclusion

In this study of patients with the monogenic disorder FGDS, we have for the first time demonstrated that increased GC-C signaling leads to prolonged CTT and changes in intestinal motility coupled to measurements of intestinal pH and circulating intestinal hormones. The lack of a plasma peak in 5-HT after a meal and prolonged colon transit time in the FGDS patients suggest motility disturbances similar to patients suffering from constipation.

## Supporting information

S1 Table Interobserver analyzes. The Limits of agreement of transit time (TT) between Observer 1 and 2 in stomach, small bowel (SBTT), colon (CTT), small bowel and colon (SBCTT) and whole gut (WGTT).
(DOCX)

S2 Table De-identified data. Datafile with results from ultrasound examinations, WMC recordings and results from hormone plasma analyzes. The data are de-idenitified.
(XLSX)
