# Supplementary material for: Prolonged intestinal transit and diarrhea in patients with an activating GUCY2C mutation
Source: PLoS One. 2017 Sep 28;12(9):e0185496. doi: 10.1371/journal.pone.0185496 (PMC5619782; doi:10.1371/journal.pone.0185496)
Supplement: S1 Table — The Limits of agreement of transit time (TT) between Observer 1 and 2 in stomach, small bowel (SBTT), colon (CTT), small bowel and colon (SBCTT) and whole gut (WGTT). (DOCX) [file pone.0185496.s001.docx]

|  | Groups | Mean difference | Lower and upper  limits of agreement | | Correlation coeffisient r | P value |
| --- | --- | --- | --- | --- | --- | --- |
| Stomach | FGDS  HC | - 0.027  0.022 | -0.25  -0.14 | 0.19  0.28 | 0.99  0.98 | < 0.001  < 0.001 |
| SBTT | FGDS  HC | 6.6  -0,27 | -21.02  -2,47 | 35.02  1,8 | 0.79  0.52 | < 0.001  < 0.01 |
| CTT | FGDS  HC | -6.6  0.02 | -35.1  -1.83 | 21.9  2.37 | 0.89  0.1 | < 0.001  < 0.001 |
| SMCTT | FGDS  HC | 0.05  0.01 | -0.56  -0.34 | 0.66  0.36 | 0.1  1 | < 0.001  < 0.001 |
| WGTT | FGDS  HC | -0.26  0.05 | -0.81  -0.15 | 1.29  0.25 | 0.1  1 | < 0.001  < 0.001 |

**Supplementary S1**. **Interobserver analyzes**  The Limits of agreement of transit time (TT) between Observer 1 and 2 in stomach, small bowel (SBTT), colon (CTT), small Bowel and colon ( SBCTT) and whole gut

(WGTT).
